# Osteoclasts affect the anti-cancer activity of NK cells

**DOI:** 10.3389/fimmu.2026.1730283

**Published:** 2026-01-30

**Authors:** Clarissa Orrico, Matteo Visca, Monica Parodi, Giulia Bertolini, Federico Davide Mussano, Riccardo Ferracini, Ilaria Roato

**Affiliations:** 1 Bone and Dental Bioengineering Lab, Centro Interdipartimentale di Ricerca (CIR) Dental School, Department of Surgical Sciences, University of Turin, Turin, Italy; 2 Department of Mechanical and Aerospace Engineering, Politecnico di Torino, Turin, Italy; 3 Immunology Operative Unit, Istituto di Ricovero e Cura a Carattere Scientifico (IRCCS) Ospedale Policlinico San Martino, Genova, Italy; 4 Unit of Epigenomics and Biomarkers of Solid Tumors, Fondazione IRCCS Istituto Nazionale dei Tumori, Milano, Italy; 5 Department of Integrated Surgical and Diagnostic Sciences, University of Genoa, Genova, Italy

**Keywords:** bone metastasis, cancer stem cell, NK cell, non small cell lung cancer, osteoclast

## Abstract

**Introduction:**

Non-small cell lung cancer (NSLSC) stem cells (CSCs) have been shown to be responsible for bone metastasis by interacting with osteoclasts (OCs) and creating an immunosuppressive environment in the bone pre-metastatic niche. Now, we investigated the interaction among OCs, IL-15-stimulated NK cells and CSCs to understand whether NK cells can interfere with the pro-metastatic crosstalk between OCs and CSCs.

**Methods:**

*In vitro* co-cultures of autologous OCs, NK cells and spheres enriched for CSC from NSCLC A549 cell line (A549/s) were set up both on plastic and bone slices, and OC activity was evaluated through quantification of tartrate-resistant acid phosphatase and of resorption area. The expression of NK cell receptors and ligands was studied through flow cytometry on NK cells, OCs and CSCs. The NK cell degranulation activity was investigated as CD107a expression.

**Results:**

The number of multinucleated/TRAP^+^ OCs and TRAP activity decreased when OCs were cultured with NK cells, and with NK cells+A549s. In presence of NK cells, A549s adhered to the bone slice surface and grew, suggesting that NK cells promote the growth of cancer cells rather than block it. Next, we studied whether NK cell phenotype and activity could be modulated by OCs and A549s. When NK cells were co-cultured with A549 cells, OCs, or with the combination of OCs and A549s, we observed a significant increase in the cytotoxic subset of CD56^+^CD16^+^ cells, a reduced expression of activating receptors (DNAM-1, NKp44) and an increased expression of inhibitory receptors (TIGIT, TIM3) on NK cells. The NK cell degranulation activity was inhibited by the presence of OCs. The addition of an anti-TIGIT antibody only partially reactivated NK cells, as indicated by a modest control of A549 cell growth, suggesting that NK cell exhaustion was induced by OCs.

**Discussion:**

All together these data show that OCs negatively affect the NK cell cytotoxic activity, allowing the growth of NSCLC CSCs. Our findings reveal a previously unrecognized role of OCs in modulating the immune microenvironment by dampening NK cell function.

## 1 Introduction

Despite significant improvements in the treatment of Non-small cell lung cancer (NSCLC), bone metastases remain the most frequent and severe deadly complication. Approximately one-third of NSCLC patients show bone lesions at diagnosis, and 40–60% will develop them during the progression of the disease (1, 2). Bone metastasis is indicated as a negative prognostic factor, particularly in NSCLC patients undergoing immunotherapy (3).Thus, it is even more important the study of the crosstalk among osteoclasts (OCs), tumor cells and immune cells, comprising the bone marrow, to identify novel therapeutic targets to prevent or limit bone metastasis development.

We previously reported that a subset of NSCLC-Cancer Stem cells (CSCs), characterized by the expression of CD133 and CXCR4, plays a key role in metastatic dissemination, including metastasis to the bone (4). These CSCs can efficiently seed metastasis at distant sites by interacting with the pre-metastatic niche components, such as osteoclasts (OCs) and they activate adenosinergic signaling, contributing to the immunosuppressive environment in the bone pre-metastatic niche (5). After reaching the bone metastatic site, tumor cells modify the microenvironment to favor their growth, and promote immune suppressor cells, such as T regulatory cells, which are normally present in bone marrow to maintain a balanced immunoreactivity (6), and myeloid derived suppressor cells (MDSCs), which can stimulate osteoclastogenesis (7), and can also directly differentiate into OCs as reported in breast cancer patients (8). Among the immune cells involved in the control and elimination of tumor cells, NK cells have a pivotal role (9). In addition to their cytotoxic function, NK cells also contribute to the regulation of both innate and adaptive immune responses. NK cells are a heterogeneous cell population, containing different subset, which display various capabilities to kill different cells. The two most well-characterized subsets of NK cells are the CD56^bright^ (CD56^++^CD16^−^) and CD56^dim^ (CD56^+^CD16^+^) subpopulations, both of which lack CD3 expression (10). CD56^bright^ NK cells are the most efficient cytokine producers but are not cytotoxic without priming by pro-inflammatory cytokines (11, 12). Conversely, the CD56^dim^ NK cells exert cytotoxic activity against infected and/or malignant cells (13). NK cell anti-tumor cytotoxic activity is mediated by a balance between activating natural cytotoxicity receptors, such as NKp30, NKp44, NKp46, NKG2D, DNAM-1, and inhibitory receptors, such as TIGIT, TIM-3, killer-cell immunoglobulin-like receptors (KIRs) (14, 15). Tumor cells often express ligands for activating and downregulate HLA class I molecules, making them suitable targets for NK cell-mediated lysis (16). However, tumors may evade immune surveillance by inducing the expression of NK cells inhibitory receptors, such as TIGIT and TIM-3 (17). Recently, we demonstrated that a highly metastatic CSC subpopulation, retaining both epithelial and mesenchymal hybrid phenotype, exhibits resistance to NK cell-mediated killing, both *in vitro* and *in vivo* (18). NK cells can also exert different effects on OCs, which express ligands for activated NK cells (19, 20).

Literature data suggest that the interaction between OCs and NK cells correlate with *in vitro* method for NK cell activation. For instance, IL-2-stimulated NK cells increase their IFN-γ secretion when co-culture with OCs (21), thus increasing their killing ability. In a humanized BLT mice model, OCs and probiotic bacteria activate IL-2-stimulated NK cells, which efficiently inhibited the growth of tumor (22). Differently, NK cells stimulated with IL-15 induced OC apoptosis in a dose-dependent manner (19), while in an *in vivo* model of rheumatoid arthritis, which is associated with an increased OC activity, IL-15-stimulated NK cells induced monocyte differentiation into OCs (20). These findings suggest that NK cells primed with different cytokines may interact with OCs and CSCs in distinct ways, either leading or not to the control of CSC growth. In the present work, we investigated the interaction among OCs, IL-15-stimulated NK cells, and NSCLC CSCs, to determine whether NK cells can interfere with the pro-metastatic crosstalk between OCs and CSCs.

## 2 Materials and methods

### 2.1 OC generation and characterization

Peripheral blood mononuclear cells (PBMCs) were obtained from 12 healthy donors’ buffy coats, through a density gradient by the Ficoll-Paque TM PLUS (Cytiva, Marlborough, USA). PBMS were in part frozen for subsequent NK separation and in part utilized for monocyte separation through CD14 microbeads and Midi MACS cells separation system (Miltenyi Biotec, Bergisch Gladbach, Germany). The purity of CD14^+^ cells (≥ 95%) was checked by flow cytometry. Cells were plated in 24- or 6- multi-well plates (Corning, NY, USA), in Alpha-Minimal Essential Medium (α-MEM, supplied by Life Technologies, NY, USA), supplemented with 10% fetal bovine serum (FBS) and 1% Penicillin-Streptomycin (Euroclone, Pero, Milan). To induce OC differentiation, recombinant human M-CSF (25 ng/ml) and RANK-L (10 ng/ml) (PeproTech, East Windsor, NJ, USA) were added every 3 days and the cultures were maintained at 37°C and 5% di CO_2_ for 8–10 days.

OC photos were obtained through an optical microscope, and tartrate-resistant acid phosphatase (TRAP) positive multinucleated cells with three or more nuclei were stained by TRAP staining kit (Cosmo Bio Co. LTD. Tokyo, Japan). The optical density of the TRAP enzymatic activity was quantified in the supernatants of OCs on the different cell culture conditions through Multiskan FC Microplate Photometer. The interaction between OCs and NK cells were monitored and registered for 72h by JulyBr Live cell movie analyzer (Nanoentek, Waltham, MA, USA).

Annexin V staining was performed on OCs following the manufacturer’s protocol (Miltenyi Biotec, Bergisch Gladbach, Germany). Briefly, Annexin V- FITC Kit allows to detect apoptotic dead cells as Annexin V positive and propidium iodide (PI) negative. All samples were acquired through MACsQuant 10 and analyzed with MACsQuantify software (Miltenyi Biotech, Bergisch Gladbach, Germany).

OCs were analyzed by flow cytometry, according to a previously published protocol (23). Briefly, OCs were detached by ACCUTASE solution (Sigma-Aldrich, St. Louis, MO, USA), and incubated with Hoechst 33342 Solution (BD Bioscence, Franklin Lakes, New Jersey, USA) for 30 minutes at 37°C. Successively, cells were washed with PBS and incubated with primary anti-human Calcitonin R antibody (CalcR IgG2a) (R&D,Systems, Minneapolis, USA) for 30 minutes at 4°C, then with the appropriate secondary IgG2a A647 and the PE-conjugated antibody mouse anti-human CD51-61 (BD Bioscence, Franklin Lakes, New Jersey, USA) for 30 minutes at 4°C. The expression of NK cell receptors’ ligands on OCs were performed using the following primary monoclonal antibodies (mAb) produced in laboratory (UOC Patologia e Immunologia, IRCCS Ospedale Policlinico San Martino, Genoa, Italy): anti-PVR (L95-IgG1), anti-Nectin2 (L14-IgG2A), while anti-ULBP1 (MAB1380, R&D System Minneapolis, USA), anti-ULBP2 (MAB1298), anti-B7H6 (MAB7144), anti-NID1 (MAB2570) were purchased from R&D System, Minneapolis, USA. The secondary antibodies used were mouse anti-IgG1 PE (1070-09, Southern Biotech, Birmingham, USA) and Alexa Fluor 647(A21240, Thermofisher Scientific, Waltham, Massachusetts, USA). All samples were analyzed using MACsQuant 10 and computed with MACsQuantify software (Miltenyi Biotech, Bergisch Gladbach, Germany).

### 2.2 Bone resorption quantification

After OCs were formed on plates (about 8–9 days), they were detached, plated on bone slices (ids, Boldon, United Kingdom) and cultured for 14 days. Then, OC resorption was evaluated through Scanning Electron Microscopy (SEM) using a ZEISS EVO 50 XVP (Oberkochen, Germany), equipped with a LaB6 source. Briefly, bone slices were undergone to alcohol dehydration (NaOH 50%, NaOH 70%, NaOH 90%, NaOH 95%, NaOH 99%), next to minimize the charge effects, the surface of the samples was previously coated with a thin gold layer (∼10 nm), then analyzed by SEM. Images of the total surface of the bone slices were analyzed to quantify the bone resorption area, by ImageJ software 1.53t.

### 2.3 Isolation, culture and characterization of NK cells

Frozen PBMCs, previously isolated and frozen as described in 2.1, were thawed to isolate NK cells through a negative selection, by NK Cell Isolation Kit (130-092-657, Miltenyi Biotec, Bergisch Gladbach, Germany), according to the manufacturer’s instructions. The overall purity of the utilized NK cells was ≥ 95%, **Supplementary Figure S1**. Cells were cultured at 200.000 cell/well in RPMI 1640 (Lonza, Basel, Switzerland) + 10% FBS + 1% Penicillin-Streptomycin, 1% Glutamine (Euroclone, Pero, Milano) supplemented with IL-15 (5 ng/ml, IL-15 ImmunoTools, Friesoythe, Germany) in U-bottom tissue culture plates (Corning, NY, USA). Medium and IL-15 were refreshed after 3 days, thus NK cells received 2 stimulations with IL-15 for up to 7 days, then they were utilized for the different co-culture conditions, without adding further IL-15. We checked the viability of NK cells by 7-AAD staining solution by Miltenyi Biotec, **Supplementary Figure S2**.

The immunophenotype of NK cells were analyzed through staining by the following primary monoclonal antibodies (mAbs), produced in laboratory (UOC Patologia e Immunologia, IRCCS Ospedale Policlinico San Martino, Genoa, Italy): AZ20 (IgG1, anti-NKp30), Z231 (IgG1, anti-NKp44), GN18 (IgG3, anti-DNAM1). We also used commercially available mAbs as CD69 PE, CD3 PercP, CD16 APC-Vio770, TIGIT (Miltenyi Biotec, Bergisch Gladbach, Germany), CD56 PE-Cy7 (anti-human Beckaman Coulter, Fullerton, CA, USA), and LEAF anti-TIM3 (345004, Biolegend, San Diego, California). The secondary antibodies used were mouse anti-IgG1 PE (1070-09, Souther Biotech, Birmingham, USA) and Alexa Fluor-647(A21240, Thermofisher Scientific, Waltham, Massachusetts, USA).

### 2.4 NSCLC CSCs formation and characterization

NSCLC cell line, A549 (ATCC, Manassas, Virginia) was plated in adherent condition in F-12K Nut Mix 1x (Gibco, Billings, MT, USA) with 10% FBS, 1% Penicillin-Streptomycin.

To obtain CSCs (A549s), A549 cells were detached by Tripsin (Euroclone, Pero, Milano), counted and plated at 10.000 cells/well in Ultra-Low Attachment 6-multi-well (Thermo Fisher Scientific, Carlsbad, CA, USA), in a serum-free stem cell medium (SCM), constituted by DMEM/F12K (Gibco, Billings, MT, USA), supplemented with B27 (Gibco, Billings, MT, USA), EGF 20 ng/mL (Sigma Aldrich, Burlington, MA, USA), bFGF10 ng/mL (PeproTech, East Windsor, NJ, USA), Heparin 2 g/mL (Stemcell Technologies, Vancouver, Canada), and 1% Penicillin-Streptomycin. After 10 days, spheroid-like structures were originated, then dissociated by ACCUTASE and analyzed by flow cytometry through the staining with the following anti-human monoclonal antibodies CD133 PE-Vio770, Nectin-2 PE, Nectin-4 Vio Bright V423 (Miltenyi Biotec, Bergisch Gladbach, Germany), CXCR4 APC (BD Bioscence, Franklin Lakes, New Jersey, USA), PVR BV510 (Biolegend, San Diego, USA).

### 2.5 Co-culture of OCs, NK cells, and A549s

Co-cultures with autologous OCs and NK cells (ratio 1:1) and with OCs+A549s+NK cells (ratio 1:1:1) were plated on flat bottom 96-well plates for 3 days. The same co-cultures were performed on bone slices in SCM with M-CSF (25 ng/mL) and RANKL (10 ng/ml) for 14 days, to allow the OC resorption activity. NK cells and A549s were co-cultured (ratio 1:1) for 3 days in SCM.

The NK degranulation activity was evaluated by CD107a staining on NK cells on day 7 (**Supplementary Figure S3**) and after 3 days of culture in medium without IL-15 or in co-cultures with A549s and OCs. Briefly, NK cells were co-cultured with A549s, OCs and both OCs and A549s (ratio of 1:1:1) in the presence of CD107a antibody (Biolegend, San Diego, USA). After 1hour, GolgiStop (BD Biosciences, Franklin Lakes, New Jersey, USA) was added to the co-cultures, and cells were incubated for 5 additional hours. Next, cells were recovered, washed once with PBS and stained for CD56 and CD16. In addition, anti-TIGIT blocking antibody (50ug/ul) (Biolegend, San Diego, USA) was added in the different co-cultures.

### 2.6 Statistical analysis

Statistical analyses were performed using GraphPad Prism version 8.0. Two-sided and paired Student’s t-test was utilized to determinate statistically significant differences between two groups. Statistical analysis among more than two groups was performed by one-way Anova with Tukey’s *post-hoc* test. Data are expressed as means ± standard deviation (SD), unless otherwise indicated. Statistical significance was defined as a *p*-value less than 0.05.

## 3 Results

### 3.1 OCs are inhibited by the interaction with NK cells and A549s

**Figure 1:**
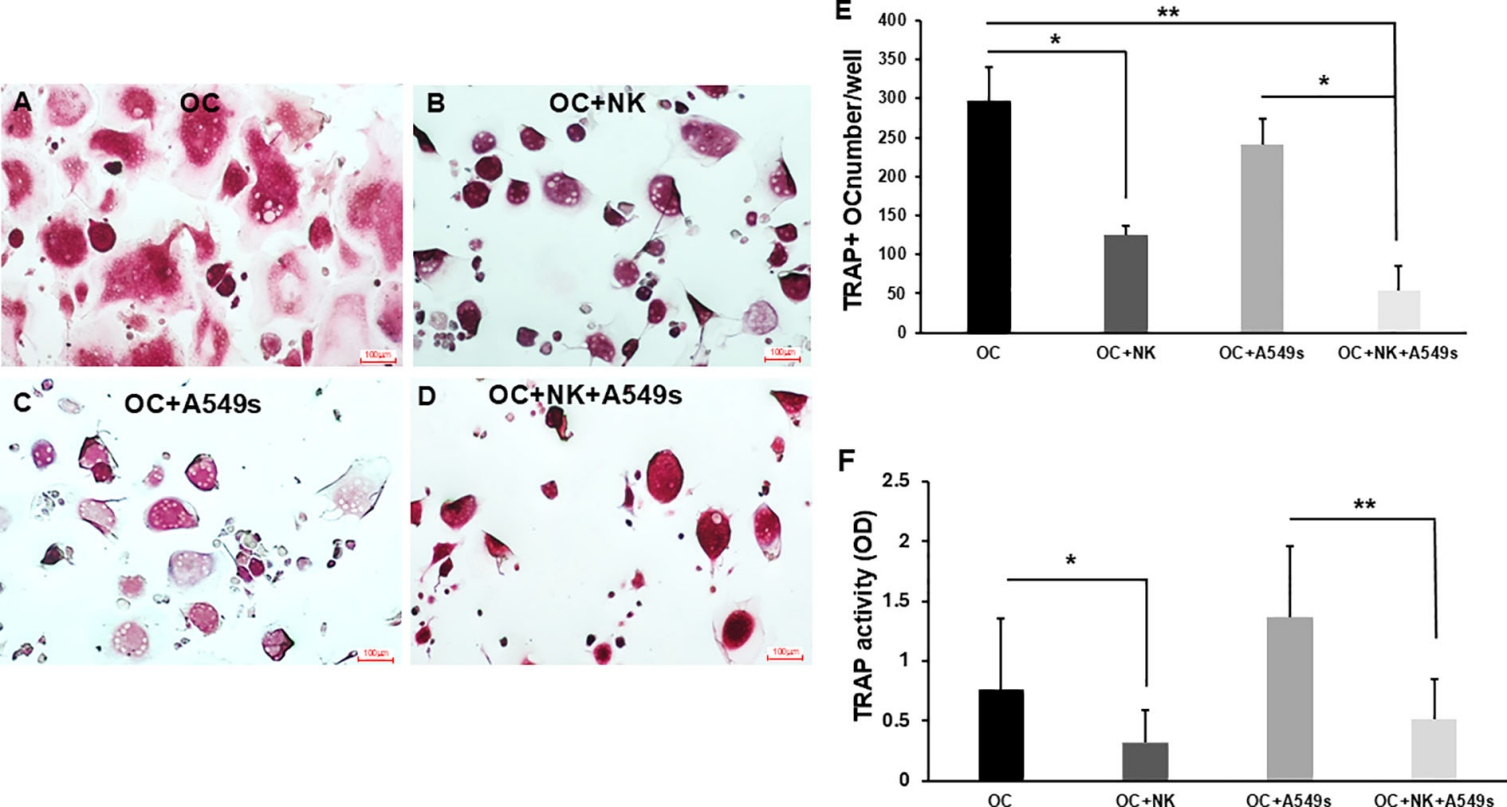
Osteoclast formation and activity. TRAP+ multinucleated OCs alone **(A)**, in co-culture with NK cells **(B)**, with A549s **(C)**, and both NK cells+A549 **(D)**, for 3 days. The graph shows the number of OCs, counted in 10 fields for well, in co-culture with NK cells, with A549s and both NK cells+A549s **(E)**. The bar plot (mean ± SD) represents the TRAP activity of OCs, in co-culture with NK cells, with A549s, and with both NK cells+A549s **(F)**, N = 5 experiments. Groups were compared using a one-way repeated measures analysis of variance with *post hoc* Tukey test (*p < 0.05; **p < 0.01).

CSCs have been widely reported to be implicated in the metastatic process. In this study, we explored the interactions between CSCs, OCs, and NK cells as fundamental players in bone metastasis process. The crosstalk among OCs, NK cells and CSCs was investigated by setting up *in vitro* co-cultures of OCs, NK cells and/or A549 spheres (A549s), enriched for CD133^+^ CSC subpopulation. After 72 hours of co-culture, we reported that multinucleated, TRAP^+^ OCs (**Figures 1A, E**) decreased when cultured with NK cells, (**Figures 1B, E**, *p < 0.05) and with both NK cells+A549s (**Figures 1D, E**, **p < 0.01).

We observed a significant decrease in OC numbers (*p* < 0.05), when co-cultured with NK cells and A549s (**Figures 1D, E**) compared to A549s alone (**Figures 1C, E**) suggesting a modulatory role of NK cells in OC viability or differentiation. Consistently, TRAP enzymatic activity was markedly decreased in OCs co-cultured with NK cells (**Figure 1F**, *p < 0.05). Notably, TRAP activity in OCs was not significantly impacted by A549s alone, but it differed significantly when cultured with both NK cells and A549s (**Figure 1F**, **p < 0.01), indicating a suppressive effect of NK cells on OC function, which may be modulated by the presence of CSC-enriched A549 spheres.

**Figure 2:**
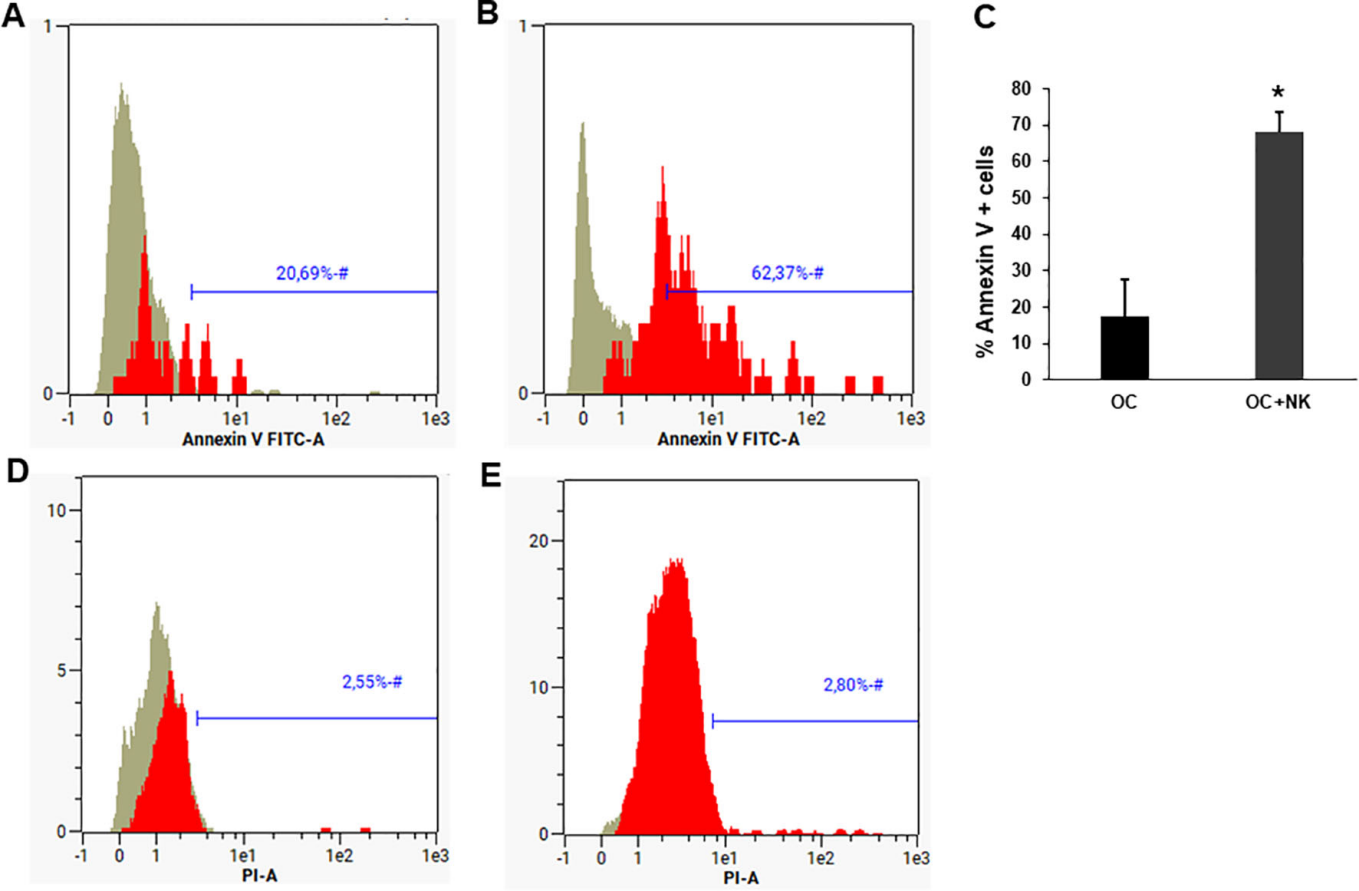
Osteoclast death mediated by NK cells. Representative histograms showing the Annexin V and PI expression respectively on OCs **(A, D)** and OCs + NK cells **(B, E)**, obtained through flow cytometric analysis. The graph (mean ± SD) shows the expression level of Annexin V for OCs alone or cultured with NK cells for 3 days, N = 3 experiments. The groups were compared using paired two-tailed t-test (*p < 0.05). The grey histogram represents the unstained cellular control, the red Annexin V+ cells.

Since we observed a reduction in OC number after co-culture with NK cells, we real-time monitored OCs and NK cell co-culture for 72h. We showed that the death of OCs was not immediate, but mainly concentrated at 48-72h, with apoptosis representing the main mechanism of cell death (Suppl. Video). Analysis of Annexin V expression in OCs cultured alone (**Figure 2A**) or with NK cells (**Figure 2B**) revealed a significant increase of Annexin V in presence of NK cells *p* < 0.01(**Figure 2C**), while PI was negative for both OCs alone (**Figure 2D**) and OCs+NK (**Figure 2E**).

### 3.2 OC TRAP activity is reduced by NK cells and A549s

**Figure 3:**
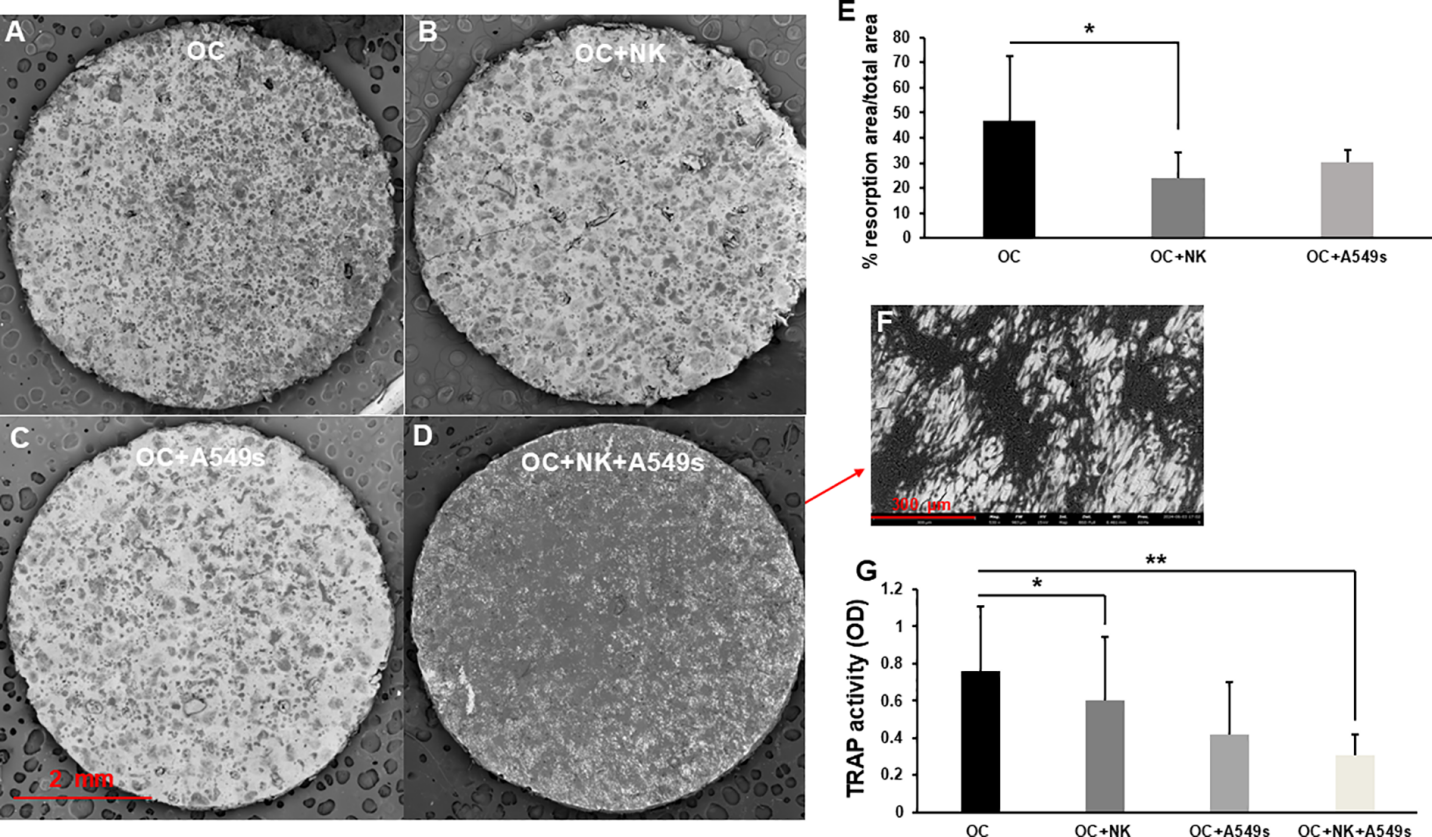
Osteoclasts activity on bone slices. SEM images show the resorption activity of OCs on bone slices **(A)**, in co-culture with NK cells **(B)**, with A549s **(C)**, and with both NK cells+A549s **(D)**, magnification 35X. The bar plot (mean ± SD) shows the quantification of resorption area of OCs, in co-culture with NK cells and with A549s **(E)**, N = 5 experiments. A549s spread on bone slice surface in presence of OCs+NK cells, at high magnification 530X **(F)**. The graph (mean ± SD) indicates the TRAP activity of OCs, in co-culture with NK cells, with A549s and both NK cells+A549s **(G)**, N = 9 experiments. Groups were compared using a one-way repeated measures analysis of variance with *post hoc* Tukey test (*p < 0.05; **p < 0.01).

After 14 days of co-culture, OC resorption activity on bone slices (**Figure 3A**), that was a measurement of OC functionality, was significantly reduced with NK cells, *p* < 0.05 (**Figures 3B, E**), whereas was not altered in co-culture with A549s compared to OCs alone (**Figures 3C-E**). Notably, in the presence of NK cells, A549s adhered to the bone slice surface and exhibited proliferative behavior (**Figures 3D, F**), thereby precluding a quantification of the resorption area under these conditions. Nonetheless, the evaluation of TRAP activity revealed a significant reduction of OC resorption activity both with NK cells alone or with A549s, *p* < 0.05 (**Figure 3G**). These findings indicate that NK cells do not exert an inhibitory effect on cancer cell proliferation but conversely, they could facilitate tumor cell growth.

### 3.3 NK cell modulation in response to OCs and A549s

**Figure 4:**
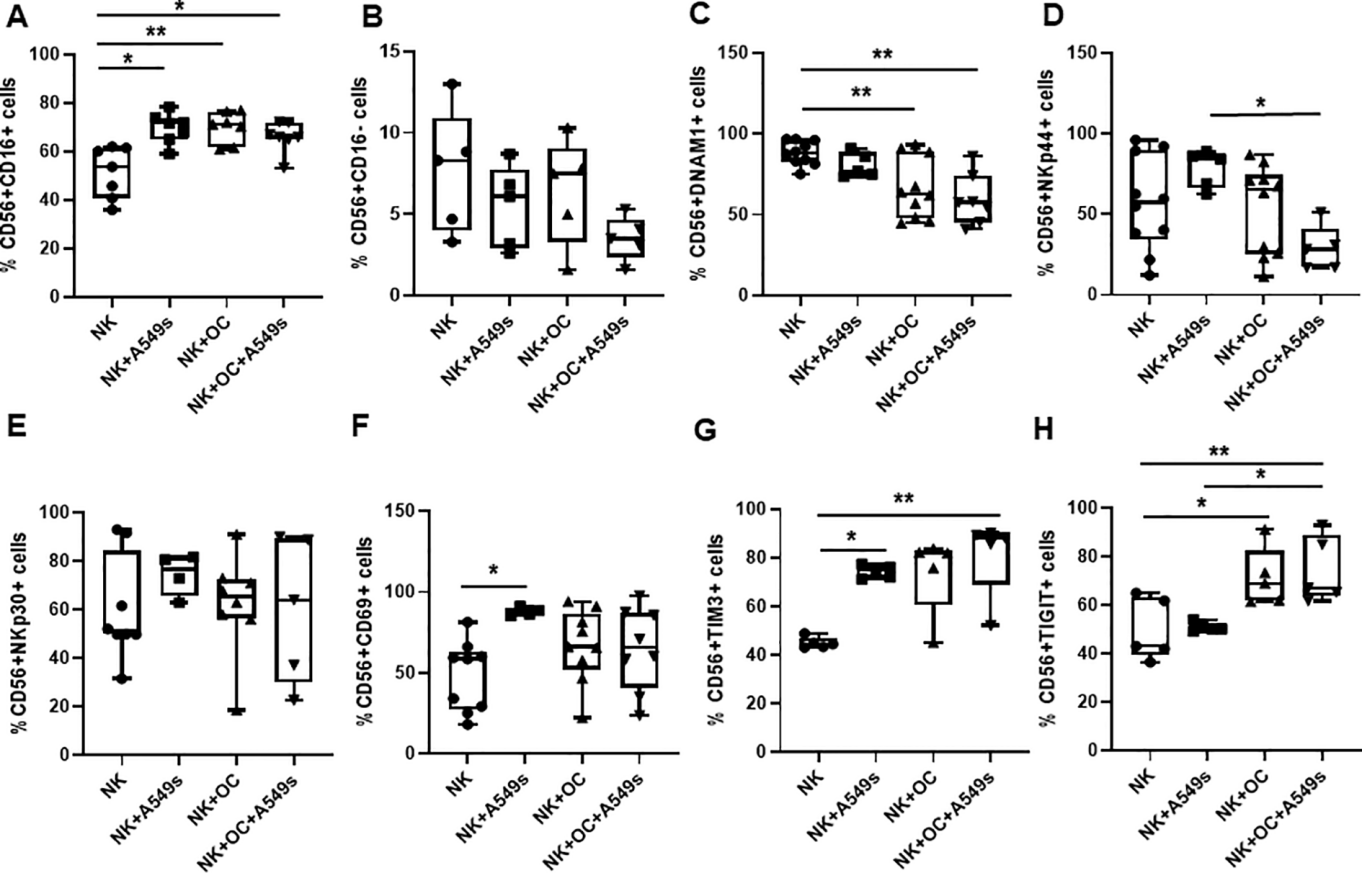
Characterization of NK cells in presence of OCs and A549s by flow cytometry. Box and whiskers plots show the % of CD56^+^CD16^+^**(A)** and CD56^++^CD16^-^ NK cells **(B)**, the expression on NK cells of NK activator receptors DNAM-1 **(C)**, NKp44 **(D)**, NKp30 **(E)**, activation marker CD69 **(F)** and of inhibitory receptors TIM3 **(G)** and TIGIT **(H)** in co-culture with A549s, OCs and both OCs+A549s. Groups were compared using a one-way repeated measures analysis of variance with *post hoc* Tukey test (*p < 0.05; **p < 0.01).

NK cells are heterogeneous: the most characterized subsets are the cytokine producer (CD56^bright^CD16^−^) and the cytotoxic (CD56^dim^CD16^+^) NK cells. We assessed whether these 2 subsets were modified after co-culture with OCs, A549s and OCs+A549s, for 3 days. We observed a significant increase of CD56^dim^ NK cell subset following co-cultured with A549s (* *p* < 0.05) OCs (**p < 0.01) and with both OCs and A549s (*p < 0.05) (**Figure 4A**) compared to NK cells alone. Conversely, the CD56^bright^ NK cell subset showed a decreasing trend in the presence of A549s and OCs+A549s (**Figure 4B**).

We further evaluated the modulation of activating (DNAM-1, NKp44, NKp30) and inhibitory receptors (TIGIT and TIM-3) on NK cells in response to co-culture with OCs and A549s. DNAM-1, a key activating receptor involved in NK cell-mediated cytotoxicity, was significantly downregulated on NK cells co-cultured with OCs or OCs/A549s (**p < 0.01) compared to control NK cells (**Figure 4C**), suggesting a potential impairment of NK cell activation. Similarly, NKp44 expression was significantly altered in NK cells co-cultured with both OCs and A549s (*p < 0.05) respect to A549s alone (**Figure 4D**), suggesting a dynamic regulation of this receptor under complex microenvironmental stimuli. Finally, we showed that NKp30 levels did not change (**Figure 4E**), whereas the activation marker CD69 was upregulated in NK cells co-cultured with A549s (**Figure 4F**), reflecting an activated phenotype in the presence of tumor cells.

Among the inhibitory receptors, we focused on TIGIT and TIM3, showing a significantly higher expression of TIM-3 by NK co-cultured with A549s (*p < 0.05), and with OCs+A549s (**p < 0.01) (**Figure 4G**). TIGIT expression was elevated in NK cells cultured with OCs and OCs+A549s compared to NK cells alone (*p < 0.05), but not with A549s alone (*p < 0.05) (**Figure 4H**). These findings suggest that OCs exert an inhibitory effect on NK cells, as evidenced by downregulation of the activating receptor DNAM-1 and upregulation of the inhibitory receptors TIM-3 and TIGIT, thereby dampening the NK cell-mediated response against A549s. Consequently, NK cells may facilitate the adhesion of A549 cells on the surface of bone slices and their proliferation (**Figure 3F**).

### 3.4 OCs modulate the expression of the ligand of NK receptors

**Figure 5:**
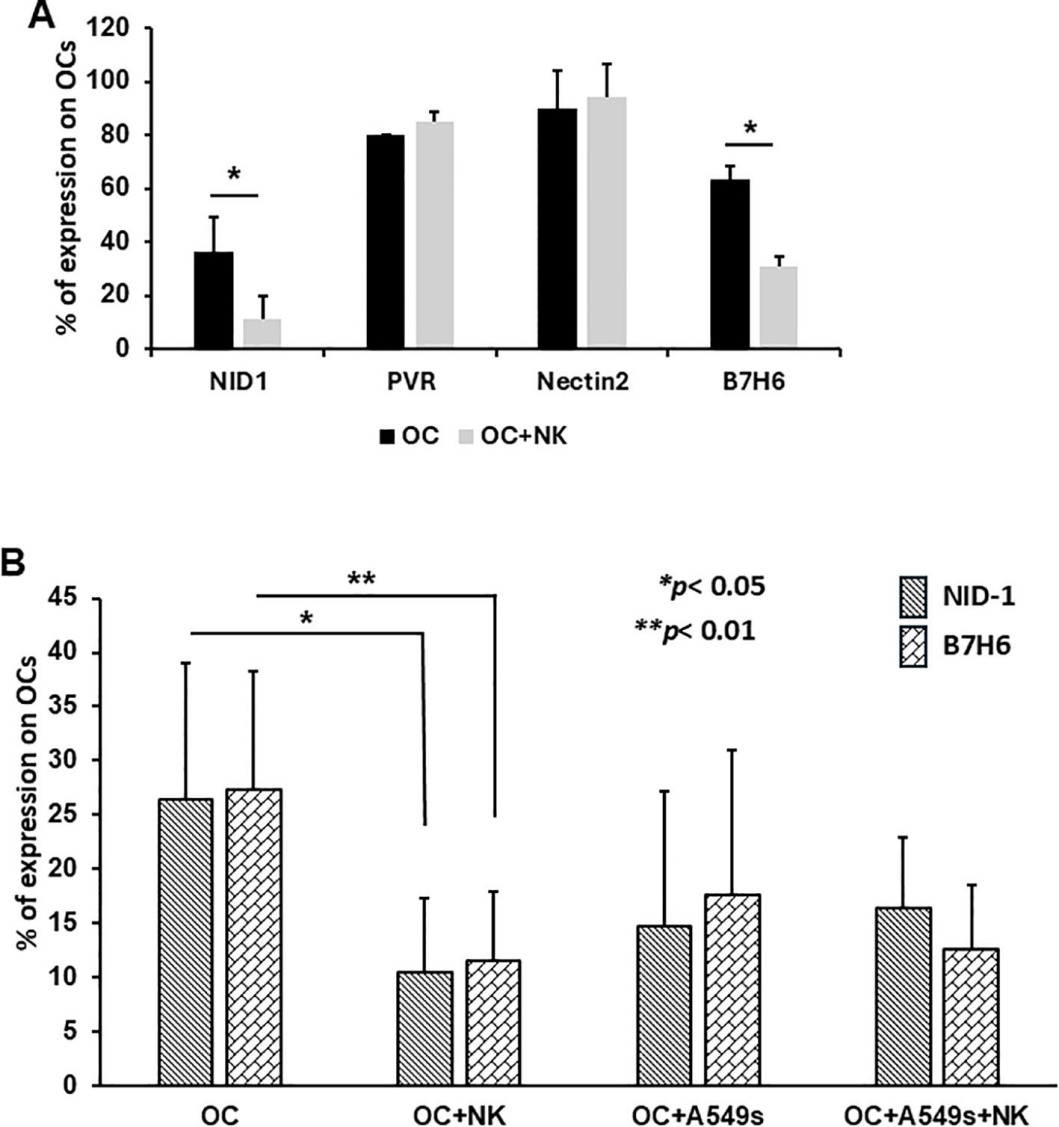
Expression of NK cell receptor ligands by OCs by flow cytometry. The graph shows the expression levels of PVR and Nectin-2, respectively ligands of DNAM-1 and TIGIT on OCs and on OC+NK cells after 3 days **(A)**. Characterization of NID-1 ligand of NKp44 and B7H6 ligand of NKp30 on OCs and on OCs+NK cells, with A549s and with both NK cells+A549s **(B)**. N = 6 experiments. Groups were compared using a one-way repeated measures analysis of variance with *post hoc* Tukey test (*p < 0.05; **p < 0.01).

Since we showed the modulation of NK cells receptors by co-cultures with OCs, we next investigated the expression on OCs of ligands for NK cell receptors on OCs. Specifically, we analyzed PVR and Nectin-2, ligands for both the activating receptor DNAM-1 and the inhibitory receptor TIGIT; NID-1, a ligand for the activating receptor NKp44; and B7H6, a ligand for NKp30, respectively controlling cytotoxic response and NK cell-mediated killing. PVR and Nectin-2 expression showed a slight but not statistically significant increase, whereas NID-1 and B7H6 expression were significantly decreased when OCs were co-cultured with NK cells (**Figure 5A**, *p < 0.05). The observed downregulation of NID-1 (**Figure 5B**, *p < 0.05) and B7H6 (**Figure 5B**, **p < 0.01) suggests a mechanism by which OCs may reduce NK cell activation, contributing to immune evasion. However, OCs co-cultured with A549 cells alone or with A549 cells and NK cells did not display any significant changes in NID-1 or B7H6, whereas the decrease of NID-1 (**Figure 5B**, *p < 0.05) and B7H6 (**Figure 5B**, **p < 0.01) was consistently confirmed in presence of NK cells alone, highlighting the modulatory impact of NK-OC interactions on ligand expression and subsequent NK cell functionality.

### 3.5 OCs inhibit NK cells degranulation activity in response to A549s

**Figure 6:**
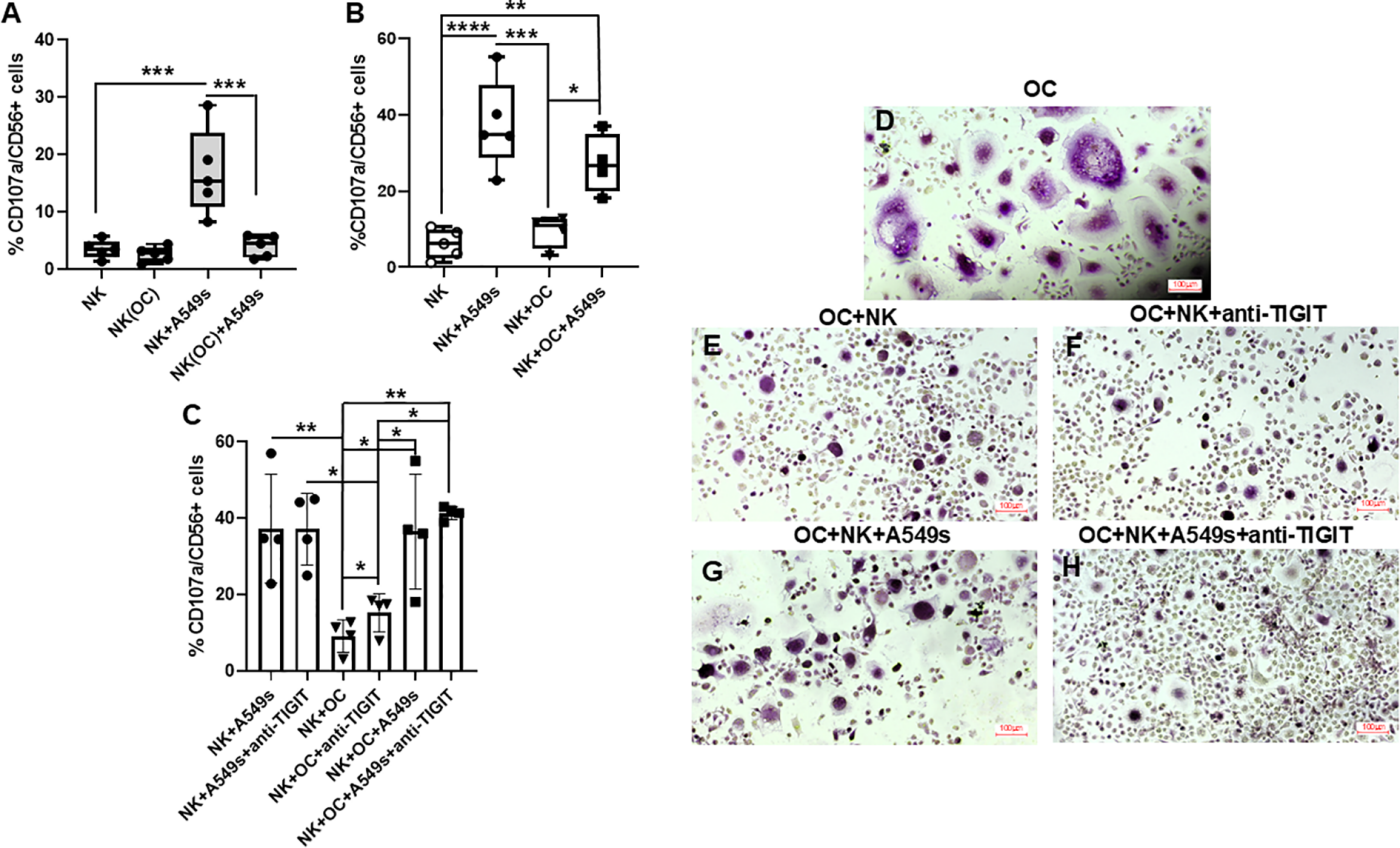
Cytotoxicity activity of NK cells in the different culture conditions. Evaluation of expression levels of CD107a by NK cells pre-conditioned w OCs, NK(OC)(ratio 1:1) or without conditioning, NK, for 3 days in response to A549s **(A)**. CD107a expression by NK cells co-culture with A549s, OCs and both A549s+OCs, without pre-conditioning **(B)**. Effect of the treatment with an anti-TIGIT antibody on NK CD107a expression, in the different co-cultures **(C)**. TRAP staining of multinucleated OCs in co-culture with NK cells w/o anti-TIGIT **(D-H)**. N = 4 experiments. Groups were compared using a one-way repeated measures analysis of variance with *post hoc* Tukey test (*p < 0.05; **p < 0.01; ***p < 0.005; ****p < 0.0001).

To assess whether OCs exert an inhibitory effect on the degranulation activity of NK cells, we co-cultured them with OCs for 72 hours, then we tested their capability to respond to A549s. NK cells degranulated against A549s as highlighted by CD107a expression (**Figure 6A**, *p < 0.05). Notably, NK cells previously cultured with OCs showed a significant reduction CD107a expression, when upon exposure to A549s compared (**Figure 6A**, *p < 0.05). Next, we evaluated the CD107a expression without pre-conditioning NK cells with OCs (**Figure 6B**). NK cells increased their degranulation in presence of A549s (****p < 0.0001) and with A549s+OCs (**p < 0.01). The level of CD107a expression was higher in NK+A549s than in NK+OCs (***p < 0.005), and likewise elevated in NK cells co-cultured with both OCs and A549 cells relative to OCs alone, *p < 0.05. Given the observed increase in TIGIT expression on NK cells in co-cultures with OCs and A549s, we investigated CD107a expression in the presence or absence of a TIGIT-blocking antibody (**Figure 6C**). The treatment with anti-TIGIT antibody did not affect degranulation in presence of A549s alone, but significantly enhanced CD107a expression when NK cells were co-cultured with OCs, *p < 0.05. NK cell activity was significantly reduced in co-culture with OCs compared to A549s (**p < 0.01), without any correlation with anti-TIGIT treatment. Interestingly, NK cell degranulation did not significantly increase in co-culture with OCs+A549s compared to OCs alone. However, the addition of anti-TIGIT antibody induced a significant increase in CD107a expression (**p < 0.01), suggesting that the presence of A549s alone is not sufficient to overcome the inhibitory effect exerted by OCs, and that the TIGIT pathway plays a critical role in the crosstalk between NK cells and OCs.

To evaluate whether NK cell degranulation correlates with a reduction in multinucleated TRAP^+^ OCs, we performed TRAP staining under different culture conditions: OCs alone (**Figure 6D**), OCs co-cultured with NK cells with or without anti-TIGIT (**Figures 6 E, F**), and OCs co-cultured with NK cells+A549s with or without anti-TIGIT (**Figures 6 G, H**). The treatment with anti-TIGIT antibody induced a marked reduction of multinucleated TRAP+ OCs (**Figures 6 F, H**), in line with the observed increase in NK cell degranulation activity.

### 3.6 CD133^+^/CXCR4^+^ stem cell subset is not significantly decreased in presence of OCs and NK cells

**Figure 7:**
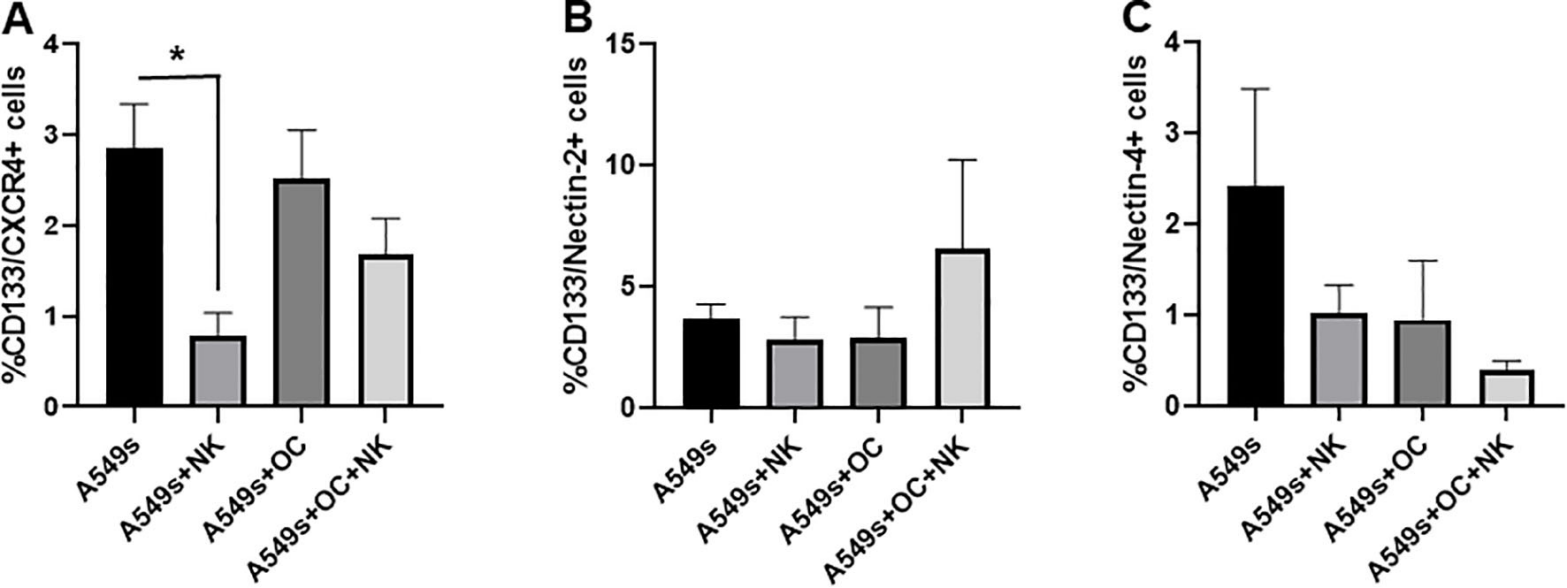
Analysis of CD133^+^/CXCR4^+^ CSC subset. Evaluation of the CSC CD133^+^/CXCR4^+^ subset of A549s, in co-culture with NK cells, OCs and with both OCs and NK cells **(A)**. Evaluation of expression levels of Nectin-2 (ligand of TIGIT and DNAM-1) **(B)** and Nectin-4 (ligand of TIGIT) **(C)** on CD133^+^ cells, in co-culture with NK cells, OCs and with NK cells+OCs **(B, C)**. N = 5 experiments. Groups were compared using a one-way repeated measures analysis of variance with *post hoc* Tukey test (*p < 0.05; **p < 0.01).

We analyzed the CD133^+^/CXCR4^+^ cell population, previously identified as the subset of metastasis-initiating cells (4), within the A549s cultured alone, or in co-culture with NK cells, OCs or both. A significant reduction of the CD133^+^/CXCR4^+^ subset was observed when A549s were co-cultured with NK cells, *p* < 0.01 (**Figure 7A**), confirming NK cell-mediated cytotoxicity against A549s. However, in the presence of NK cells+OCs, this cell population was not significantly reduced, suggesting that the immunosuppressive effect exerted by OCs may enable A549s to evade NK cell killing.

We further analyzed on CD133^+^ A549s the expressions of Nectin-2, Nectin-4 and PVR, which are the ligands of the DNAM-1 and TIGIT, the NK cell receptors significantly modulated in the different co-cultures.

We further analyzed on CD133^+^ A549s the expression of Nectin-2, PVR and Nectin-4, which is a ligand expressed by tumor cells. Nectin-2 expression showed a slight increase in A549s co-cultured with NK cells+OCs, but it was not statistically significant (**Figure 7B**). In contrast, Nectin-4 expression displayed a downregulation in CD133^+^ A549s when cultured with NK cells or OCs. PVR expression was very low in the CD133^+^ subset without differences in the different culture conditions (data not shown).

These findings may partially explain the lack of enhanced NK cell degranulation observed following anti-TIGIT treatment in co-cultures with NK cells+A549s or with OCs+A549s. In particular, the absence of significant modulation in TIGIT ligands on A549s suggests a limited contribution of the TIGIT axis in these settings, reinforcing the idea that OCs, rather than A549s, are the primary drivers of NK cell inhibition via TIGIT engagement.

## 4 Discussion

In this study, we show that IL-15-stimulated NK cells induce a reduction in the number and the activity of OCs, in line with findings previously published by Feng et al. (19). While the presence of CSCs (A549s) alone do not significantly affect OC activity. Our results reveal an inhibitory effect exerted by OCs on NK cells: although A549s typically stimulate NK cell cytotoxicity, this effect is inhibited in the presence of OCs. Indeed, in our *in vitro* system, NK cells effectively killed A549s. However, upon contact with OCs, NK cells lost this cytotoxic activity, allowing A549s to spread and colonize bone slice surfaces more extensively than when cultured with OCs alone. This observation intriguingly suggests that NK cells might indirectly promote A549 cell growth under these conditions.

An immunosuppressive role of OCs was previously reported in multiple myeloma, where OCs directly inhibit the anti-tumor T cell response (24). Conversely, OCs were shown to activate NK cells against tumor in humanized BLT mice, although NK cells were not stimulated with IL-15 (22). Notably, NSCLC tumor microenvironment locally impairs NK cells, and intra-tumoral NK cells dramatically reduced their receptors and lost their ability to degranulate and to release IFN-γ, thereby supporting cancer progression. Moreover, cell-to-cell contact has been reported to reduce NK cell tumor-killing activity (25). Taking together, these data suggest that in our system, NK cells probably receive a dual inhibitory signal from both OCs and A549s, leading to impaired anti-tumor activity.

This study aims to investigate the crosstalk between OCs, NSCLC cancer stem cells (CSC), and NK cells, demonstrating that OCs negatively impact NK cell cytotoxic activity, facilitating the growth of lung CSCs. Previous studies have shown that cancer cells can stimulate osteoclast activity; for example, breast cancer cell growth in bone is supported by OCs, which produce high levels of glutamine that sustain metastatic cells (26). Additionally, we have previously reported that CSCs derived from NSCLC can promote osteoclastogenesis when co-cultured with OC precursors (5).

The responsiveness of NK cells depends on an integration of both stimulatory/inhibitory signals and co-inhibitory signals (27). Therefore, we analyzed the presence of CD56^dim^ and CD56^bright^ NK cell subsets, as well as the expression of activating and inhibitory receptors on NK cells under different co-culture conditions. The CD56^dim^ NK cell subset increased in the co-culture of NK cells with A549s, OCs or A549s+OCs whereas the bright CD56^++^CD16^-^ subset showed a decreasing trend in the co-culture with A549s+OCs. Since OC death was observed several days after co-culture with NK cells, we hypothesize that CD56^dim^ NK cells, endowed with mature and cytotoxic profile (28, 29), mediate cytotoxic activity and induce apoptosis, also according to Feng et al. (19). Indeed, apoptotic OC death induced by NK cells was confirmed by increased Annexin V expression on OCs in co-culture. NK cells may induce apoptosis of OCs as part of their role in immunosurveillance and osteoimmunological regulation. Activated or differentiating OCs can express stress-induced ligands and/or display reduced MHC class I expression, rendering them susceptible to NK cell recognition and cytotoxicity by releasing of cytotoxic granules containing perforin and granzymes, as well as through death receptor–mediated pathways (e.g., Fas/FasL and TRAIL) (19). Based on our experimental design, the observed apoptotic effects are consistent with a direct cytotoxic activity of NK cells against OC cells, as visible by monitoring the interaction between OCs and NK cells. This interaction may represent a physiological mechanism to limit excessive OC activity and maintain bone homeostasis, particularly under inflammatory or pathological conditions.

We observed a down-regulation of DNAM-1 (the receptor for PVR, Nectin 2) on NK cells co-cultured either OCs or with A549s+OCs, while the inhibitory receptors TIM-3 and TIGIT were up regulated. Additionally, NKp44 (the receptor for NID-1) expression was reduced on NK cells co-cultured with A549s+OCs, whereas no significant change in NKp30 expression was detected. This simultaneous downregulation of activating receptors and upregulation of inhibitory receptors in the presence of OCs may reflect an attempt by OCs to evade NK cell-mediated killing. Indeed, mature OCs express different ligands for NK cell receptors (19), and we investigated whether their modulation could occur during the different co-culture conditions. We observed a significant decrease in NID-1 and B7H6 (NKp44 and NKp30, respectively) expression by OCs, following co-culture with NK cells alone or combined with A549s. This reduction supports the observed decrease in activating receptors on NK cells, leading to diminished cytotoxic activity. Correspondingly, CD107a expression was significantly increased when NK cells were co-cultured with A549s, but this expression was reduced when NK cells were pre-conditioned with OCs, suggesting an inhibitory effect exerted by OCs on NK cells. Furthermore, in co-cultures of NK cells with A549s and OCs, CD107a expression was significantly higher than in NK cells+OCs, but not significantly different from NK cells+A549s. This suggests that the presence of A549s alone cannot counteract the inhibitory influence of OCs on NK cell activity. Consequently, in the presence of OCs, NK cell–mediated cytotoxicity against A549s remains only partially effective.

Since we reported an increased TIGIT expression by NK cells co-cultured with OCs, we investigated the potential effects of a monoclonal anti-TIGIT antibody on NK cell activity across the different co-culture conditions. A growing interest in TIGIT blockade has emerged across several clinical trials aimed at enhancing immunotherapeutic treatment for cancers (30–32). However, its role in controlling bone metastases remains unexplored. Interestingly, we found that anti-TIGIT treatment induced NK cell-mediated killing of OCs, while NK degranulation did not significantly increase in the co-culture with OCs+A549s compared to co-culture with OCs alone. Only the addition of anti-TIGIT induced a significant increase in CD107a expression, suggesting that OCs exert a suppressive effect on NK cells by TIGIT. TIGIT is an inhibitory receptor expressed on NK and T cells. Its expression is known to be upregulated in tumor microenvironment, contributing to immune cell exhaustion and limiting anti-tumor responses, in particular in NK cells (33, 34). TIGIT competes with the activating receptor DNAM-1 for binding to the ligands PVR and Nectin-2, which we detected on OCs alone and co-cultured with NK cells. We observed low levels of expression of PVR and Nectin-2 on A549s, which may represent an immune-escape mechanism allowing A549s to evade DNAM-1-mediated NK cell activation. We also assessed Nectin-4 expression, given that Nectin-4 is overexpressed in several tumors (35), is associated with cancer progression (36), even in NSCLC (37), and considering its interaction with TIGIT in inhibiting NK cell cytotoxicity (38). A549s expressed low levels of Nectin-4, and its expression showed a decreasing trend across the co-cultures. This may explain why anti-TIGIT treatment did not enhance NK cell responses against A549 cells.

Our findings are based on an *in vitro* model of crosstalk among autologous OCs, NK cells and CSCs, which still require validation *in vivo*, and we are planning to do it. Moreover, we are aware that we only tested A549 cells and no other NSCLC cell lines, but we chose A549 since they are a recognized model of osteotropic NSCLC cell line, and we previously tested it in an *in vivo* models of bone metastases (39).

These novel and previously unreported results offer new insights into the role of OCs in modulating the immune microenvironment by limiting NK cell activity, which are a key player in the immune system fight against cancel cells.

## Data Availability

The raw data supporting the conclusions of this article will be made available by the authors, without undue reservation.
