# Supplementary material for: Osteoclasts affect the anti-cancer activity of NK cells
Source: Front Immunol. 2026 Jan 30;17:1730283. doi: 10.3389/fimmu.2026.1730283 (PMC12901471; doi:10.3389/fimmu.2026.1730283)
Supplement: Supplementary file 1 [file Presentation1.pptx]

## Slide 1
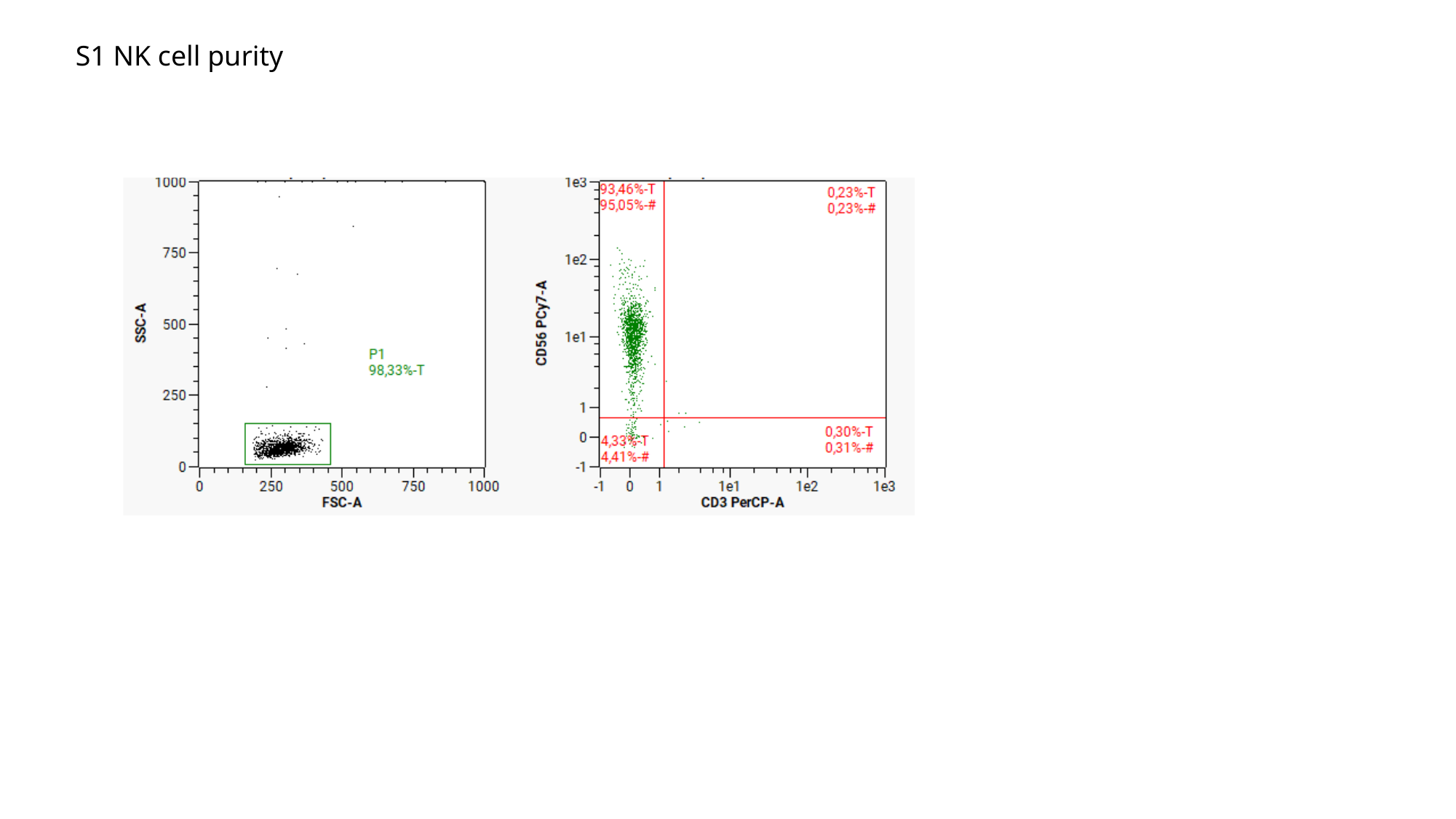

S1 NK cell purity

## Slide 2
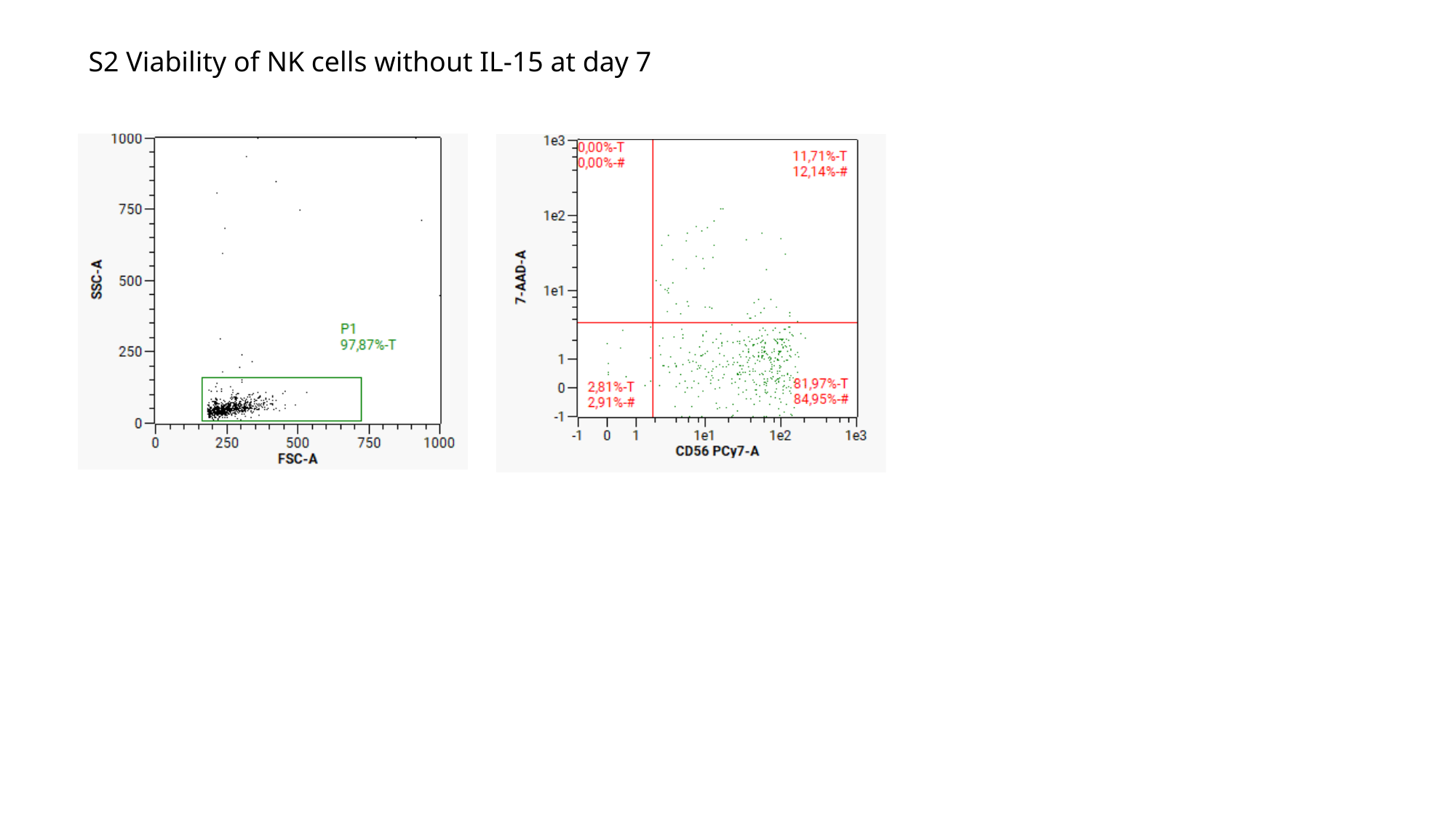

S2 Viability of NK cells without IL-15 at day 7

## Slide 3
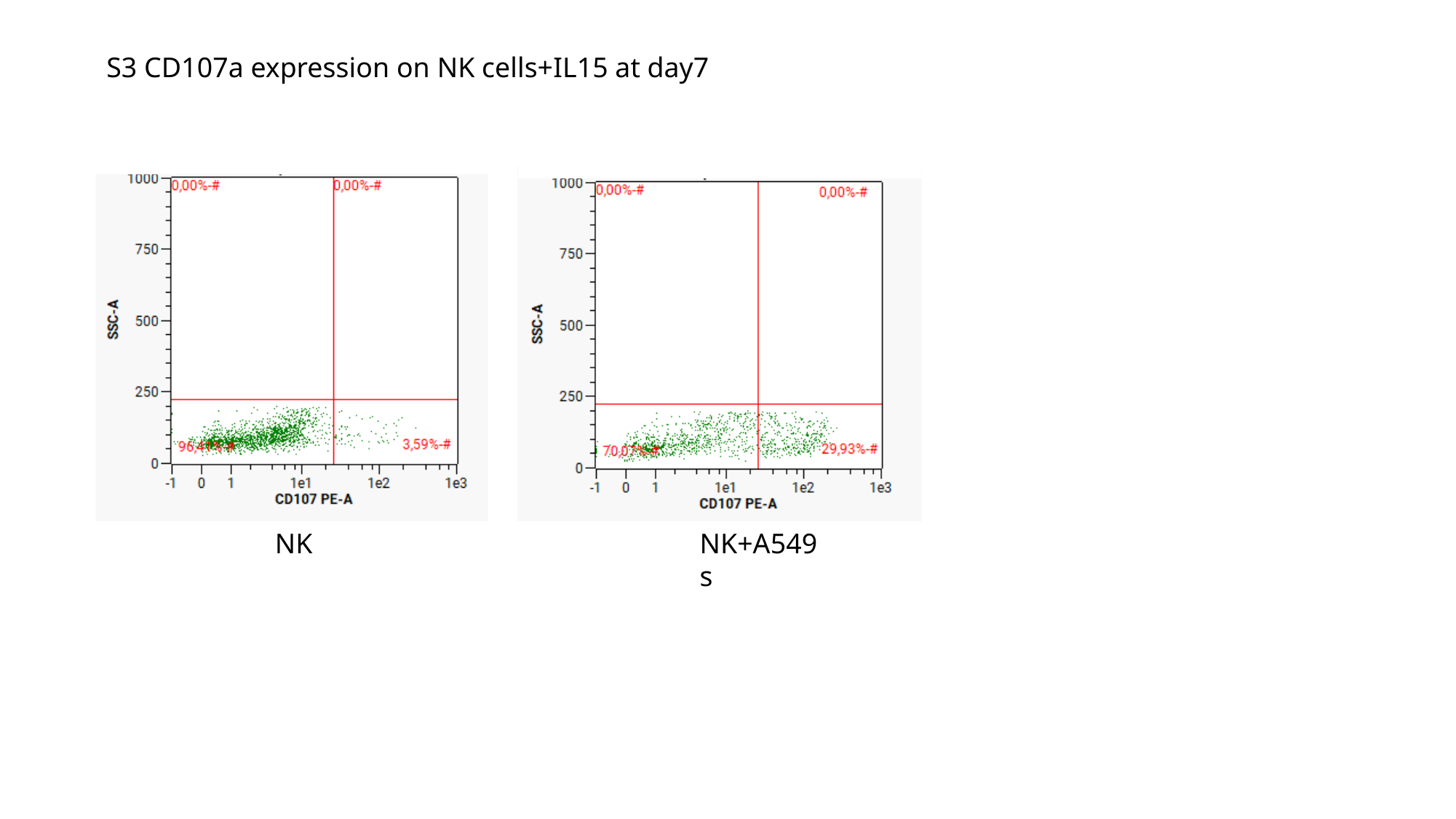

S3 CD107a expression on NK cells+IL15 at day7
NK
NK+A549s
